# A moving observer in a three-dimensional world

**DOI:** 10.1098/rstb.2015.0265

**Published:** 2016-06-19

**Authors:** Andrew Glennerster

**Affiliations:** School of Psychology and Clinical Language Sciences, University of Reading, Reading RG6 7BE, UK

**Keywords:** three-dimensional vision, moving observer, stereopsis, motion parallax, scene representation, visual stability

## Abstract

For many tasks such as retrieving a previously viewed object, an observer must form a representation of the world at one location and use it at another. A world-based three-dimensional reconstruction of the scene built up from visual information would fulfil this requirement, something computer vision now achieves with great speed and accuracy. However, I argue that it is neither easy nor necessary for the brain to do this. I discuss biologically plausible alternatives, including the possibility of avoiding three-dimensional coordinate frames such as ego-centric and world-based representations. For example, the distance, slant and local shape of surfaces dictate the propensity of visual features to move in the image with respect to one another as the observer's perspective changes (through movement or binocular viewing). Such propensities can be stored without the need for three-dimensional reference frames. The problem of representing a stable scene in the face of continual head and eye movements is an appropriate starting place for understanding the goal of three-dimensional vision, more so, I argue, than the case of a static binocular observer.

This article is part of the themed issue ‘Vision in our three-dimensional world’.

## 1. Introduction

Many of the papers in this issue consider vision in a three-dimensional world from the perspective of a stationary observer. Functional magnetic resonance imaging, neurophysiological recording and most binocular psychophysical experiments require the participant's head to be restrained. While this can be useful for some purposes, it can also adversely affect the way that neuroscientists think about three-dimensional vision, since it distracts attention from the more general problem that an observer must solve if they are to represent and interact with their environment as they move around. It is logical to tackle the general problem first and then to consider static binocular vision as a limiting case.

Marr famously described the problem of vision as ‘knowing what is where by looking’ [1]. But ‘where’ is tricky to define. It requires a coordinate frame of some kind and it is not obvious what this (or these) should be. Gibson [2] emphasized the importance of ‘heuristics’ by which visual information could be used to control action, such as the folding of a gannet's wings [3], without relying on three-dimensional representations and sometimes he appeared to deny the need for representation altogether. However, it is evident that animals plan actions using representations, for example when they retrieve an object that is currently out of view, but the form that these representations take is not yet clear. I will review the approach taken in computer vision, since current systems based on three-dimensional reconstruction work very well, and in the visual system of insects, since they use quite different methods from computer vision systems and yet operate successfully in a three-dimensional environment. Current ideas about three-dimensional representation in the cortex differ in important ways from either of these because biologists hypothesize intermediate representations between image and world-based frames; I will discuss some of the challenges that such models face and describe an alternative type of representation based on quite different assumptions.

## 2. Possible reference frames

### (a) Computer vision

Computer vision systems are now able to generate a representation of a static scene as the camera moves through it and to track the 6 d.f. movement of the camera in the same coordinate frame. This ‘simultaneous localization and mapping’ (SLAM) can be done in real time [4] with multiple moving objects [5] and even when nothing in the scene is rigid [6]. Nevertheless, in all these cases, the rotation and translation of the camera are recovered in the same three-dimensional frame as the world points. The algorithms are quite unlike those proposed in the cortex and hippocampus since the latter involves a sequence of transformations from eye-centred to head-centred and then world-centred frames (see §2c). In computer vision, the scene structure and camera motion over multiple frames are generally recovered in a single step that relies on the assumption of a stable scene [7].

**Figure 1.**
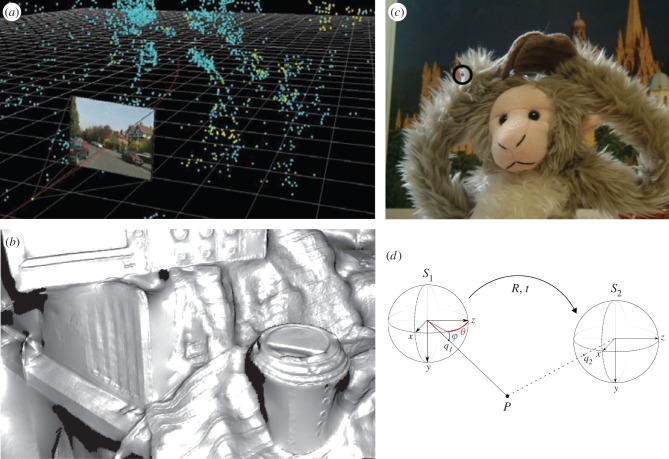
Computer vision approaches. (*a*) Early photogrammetry methods tracked features across a sequence of frames and calculated a set of three-dimensional points and a camera path that would best explain the tracks (Image courtesy of Oxford Metrics (OMG plc), [10]). (*b*) ‘Dense SLAM’ now achieves the same result but for a very dense reconstruction of surfaces and is done in real time (© Reprinted from Newcombe *et al*. [11] with permission from IEEE). (*c*) Sometimes it is very difficult to calculate the three-dimensional structure of a scene, as here, and for many purposes solutions that avoid three-dimensional reconstruction are optimal (in this case, synthesising a novel view given several input views; © Reprinted from Fitzgibbon *et al*. [12] with permission from IEEE). (*d*) Recent approaches to SLAM incorporate views at certain locations (*S*_1_ and *S*_2_ here, called ‘keyframes’) and store these, along with the rotation and translation required to move between them, as a graph (Reprinted from Twinanda *et al*. [13] with permission from the authors). (Online version in colour.)

Early three-dimensional reconstruction algorithms generally identified small, robust features in the input images that can be tracked reliably across multiple frames [7–9]. The output is a ‘cloud’ of three-dimensional points in a world-based frame, each point corresponding to a tracked feature in the input images (e.g. figure 1*a*). Modern computer vision systems can carry out this process in real time giving rise to a dense reconstruction (figure 1*b*) and a highly reliable recovery of the camera pose [4,11]. Many SLAM algorithms now also incorporate an ‘appearance-based’ element, such as the inclusion of ‘keyframes’ where the full video frame or omnidirectional view is stored at discrete points along the path which aids re-orientation when normal tracking is lost and helps with ‘loop-closure’ [13,14]. A ‘pose graph’ describes the relationship between the keyframes (figure 1*d*). Nevertheless, the edges of the graph are three-dimensional rotations and translations and there are local three-dimensional representations at each node. More recent examples abandon the pose graph and demonstrate how it is possible to build a detailed, three-dimensional, global, world-based representation for large-scale movements of the camera [15].

On the other hand, some computer vision algorithms have abandoned the use of three-dimensional reference frames to carry out tasks that, in the past, might have been tackled by building a three-dimensional model. For example, Rav-Acha, Kohli, Rother and Fitzgibbon [16] show how it is possible to add a moustache to a video of a moving face captured with a hand-held camera. In theory, this could be achieved by generating a deformable three-dimensional model of the head, but the authors' solution was to extract a stable texture from the images of the face (an ‘unwrap mosaic’), add the moustache to that and then ‘paste’ the new texture back onto the original frames. The result appears convincingly ‘three-dimensional’ despite the fact that no three-dimensional coordinates were computed at any stage. Closely related image-based approaches have been used for a localization task [17]. In the movie industry and in many other applications, the start and end points are images, in which case an intermediate representation in a three-dimensional frame can often be avoided. Another case is image interpolation, using images from two or more cameras. In theory, this can be done by computing the three-dimensional structure of the scene and projecting points back into a new, simulated camera. But for some objects, like the fluffy toy in figure 1*c*, this is hard. The best-looking results are obtained by, instead, optimizing for ‘likely’ image statistics in the simulated scene, using the input frames to determine these statistics [12] and once again avoiding the generation of a three-dimensional model. A very similar argument can be applied to biology. Both the context for and the consequence of a movement are a set of sensory signals [18], so it is worth considering whether the logic developed in computer graphics might also be true in the brain, i.e. that a non-three-dimensional representation might do just as well (under most conditions) as a putative internal three-dimensional model.

### (b) Image-based strategies

It is widely accepted that animals achieve many tasks using ‘image-based’ strategies, where this usually refers to the control of some action by monitoring a small number of parameters such as the angular size of an object on the retina and/or its rate of expansion as the animal moves. Even in insects, these strategies can be quite sophisticated. Cartwright and Collett [19] showed how bees remember and match the angles between landmarks to find a feeding site and, when the size of a landmark is changed, they alter their distance to match the retinal angle with the learned size. Ants show similar image-based strategies in returning to a place or following a route [20,21]. Equally, it is widely accepted that many simple activities in humans are probably achieved using image-based rules, such as the online correction of errors in reaching movements [22,23], and the fixation locations chosen by the visual system during daily activities often make it particularly easy for the brain to monitor visual parameters that are useful for guiding action, e.g. fixating a target object and bringing the image of the hand towards the fovea [24]. There is evidence for many tasks being carried out using simple strategies including cornering at a bend [25], catching a fly-ball [26] or timing a pull shot in cricket [27] and there is a long history of using two-dimensional image-based strategies to control robots [28].

**Figure 2.**
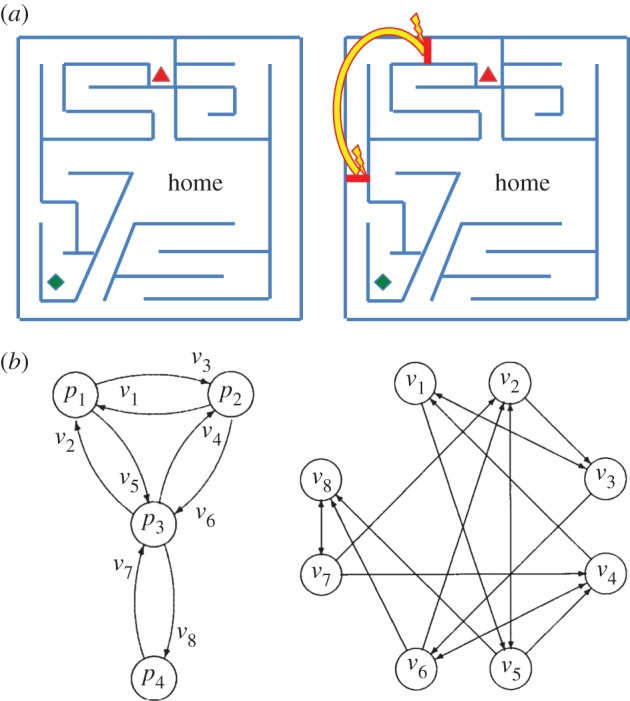
Non-Euclidean representations. (*a*) In an experiment by Schnapp and Warren [31], participants explored a virtual environment that either corresponded to a fixed Euclidean structure (left) or something that was not Euclidean (right) because, in this case, participants were transported through a ‘wormhole’ between the locations marked on the map by red lines but the views from these two locations were identical so there was no way to detect the moment of transportation. In the wormhole condition, the relative location of objects has no consistent geometric interpretation (sketch of virtual maze adapted from Schnapp and Warren [31]). (*b*) Four places, *p*_1_–*p*_4_, are shown as nodes in a graph whose edges are the views from each place. The views themselves can be described as a graph (right). In this case, the edges are actions (rotations on the spot or translations between places). (© Reprinted from Gillner & Mallot [30] with permission from MIT Press.) (Online version in colour.)

Movements take the observer from one state to another (hand position, head position, etc.) and hence, in the case of visually guided movements, from one set of image-based cues (or sensory context) to another. Image-based strategies are *ad hoc,* unlike a ‘cognitive map’ whose whole purpose is to be a common resource available to guide many different movements [29]. But image-based strategies require *some* sort of representation that goes beyond the current image. Gillner and Mallot [30], for example, have measured the ability of participants to learn the layout of a virtual town, navigate back to objects and find novel shortcuts. They suggested that people's behaviour was consistent with them building up a ‘graph of views’, where the edges are actions (forward movement and turns) and the nodes are views (figure 2*b*). Similarly, data by Schnapp and Warren (in an abstract form, [31,32]) have tested participants' ability to navigate in a virtual reality environment that does not correspond to any possible metric structure. It contains ‘wormholes’ that transport participants to a different location and different orientation in the maze without them being aware that this has happened (see figure 2*a*). Because they are translated and rotated as they move through the wormhole, no coherent (wormhole-free) two-dimensional map of the environment is possible. The fact that participants do not notice and can perform navigation tasks well suggests that they are not using a cognitive map but their behaviour is consistent with a topological representation formed of a graph of views.

Some behaviours are more difficult to explain within a view-based or sensory-based framework. Path integration by ants provides a good example: they behave as if they have access to a continually updated vector (direction and distance) that will take them back to home. This can be demonstrated by transporting an ant that has walked away from its nest and releasing it at a new location [33]. Müller and Wehner do not use this evidence to propose a cognitive map in ants but, instead, argue that the pattern of systematic errors in the path integration process suggests that the ants are using a simple mechanism that is closely analogous to homing by matching a ‘snapshot’, i.e. a classic image-based strategy. Nevertheless, for some tasks it is difficult to imagine image-based strategies: ‘Point to Paris!’, for example. People are not always good at these tasks and they often require considerable cognitive effort. It is not yet clear whether, in these difficult cases, the brain resorts to building a three-dimensional reconstruction of the scene or whether there are yet-to-be-determined view-based approaches that could account for them.

### (c) Cortical representations

**Figure 3.**
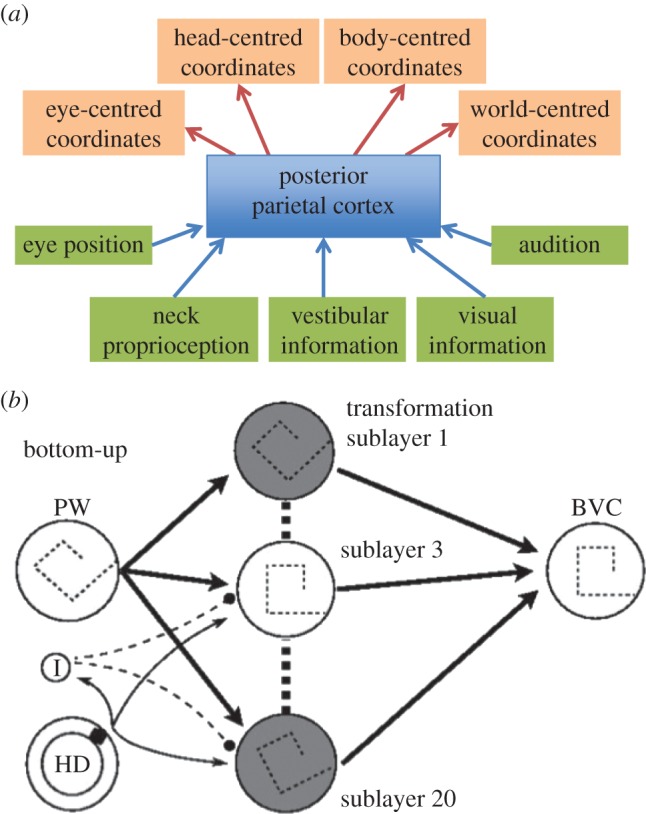
Putative neural representations of three-dimensional space. (*a*) This diagram, adapted from Andersen *et al.* [34], reflects a common assumption that parietal cortex in primates transforms sensory information of different types into three-dimensional representations of the scene in a variety of different ego-centric coordinate frames. (*b*) Byrne *et al.* [36] propose a mechanism for transforming an ego-centric representation into a world-based one using the output of head-direction cells. A set of identical populations of neurons (20 in this example, nominally in the retrosplenial cortex) each encode a repeated version of the scene but rotated by different amounts, on the basis of an ego-centric input from parietal cortex (PW). A signal from head-direction (HD) cells could ‘gate’ the information and so ensure that the output to boundary vector cells (BVC), which are hypothesised to exist in parahippocampal cortex, is maintained in a world-centred frame. (Copyright © 2007 by the American Psychological Association. Reproduced with permission.) (Online version in colour.)

It is often said that posterior parietal cortex represents the scene in a variety of coordinate frames [34,35], as shown in figure 3*a*. The clearest case for something akin to a three-dimensional coordinate frame is in V1. Here, receptive fields are organized retinotopically and neurons are sensitive to a range of disparities at each retinal location (e.g. [37]). Described in relation to the scene, this amounts (more or less) to a three-dimensional coordinate frame centred on the fixated object. In theory, a rigid rotation and translation could transform the three-dimensional receptive fields in V1 into a different frame, e.g. one with an origin and axes attached to the observer's hand. If this were the case, one would expect a very rapid and quite complex re-organization of receptive fields in posterior parietal cortex as the hand translated or rotated. But that would be substantially more complex than the type of operations that have been proposed up to now. For example, ‘gain fields’ demonstrate that one parameter, such as the position of the eyes in the head, can modulate the response of neurons to visual input [38,39]. In some cases, operations of this type can give rise to receptive fields that are stable in a non-retinotopic frame (e.g. [40]).

Beyond posterior parietal cortex, a further coordinate transformation is assumed to take place to bring visual information into a world-based frame. Byrne *et al.* [36] describe steps that would be required to achieve this (figure 3*b*). An ego-centred representation, assumed to come from parietal cortex, would need to be duplicated many times over so that a signal from a head direction cell could ‘gate’ the information passing to ‘boundary vector cells’ (BVC) in parahippocampal gyrus. This putative mechanism deals with rotation. A similar duplication would presumably be required to deal with translation.

Anatomically nearby, but quite different in their properties, ‘grid cells’ in the dorsocaudal medial entorhinal cortex provide information about a rat's location as it moves [41]. The signals from any one of these neurons are highly ambiguous about the rat's location. Three grid cells at each of three spatial scales could, in theory, signal a very large number of locations, just as nine digits can be used to signal a million different values, but the readout of these values to provide an unambiguous signal that identifies a large number of different locations would be difficult [42] especially if realistic levels of noise in the grid cells were to be modelled. Instead, arguments have been advanced that ‘place’ cell receptive fields are not built up from ‘grid’ cell input but that, instead, information from place and grid cells complement one another [43,44]. In relation to this volume, which is about vision in a three-dimensional world, it is relevant to note that grid cells are able to operate very similarly in the dark and the light [41], so the visual coordinate transformations discussed above are clearly not necessary to stimulate grid cell responses. Indeed, some have argued that grid cells play a key role in navigation only in the dark [44].

### (d) Removing the origin

Instead of representing space as a set of receptive fields, e.g. with three-dimensional coordinates defined by visual direction and disparity in V1 as discussed in §2c, an alternative is that it could be represented by a set of sensory contexts and the movements that connect these (like a graph, as discussed in §§2a and 2b). Similarly, shape and slant could be represented by storing the propensity of a shape to deform in particular ways as the observer moves—again, it is the linking of sensory contexts via motor outputs that is important [45]. The idea is not to store all motion parallax as an observer moves. Any system that did this would be able (in theory) to compute the three-dimensional structure of the scene and the trajectory of the observer. Instead, the idea is that what is stored is some kind of ‘summary’ that is useful for action despite being incomplete. I will refer throughout this section to *movement* of the observer (or optic centre), but the key is that the scene is viewed from a number of different vantage points. A limiting case is just two vantage points, including a static binocular observer, but the principles should apply to a much wider range of eye and head movements.

**Figure 4.**
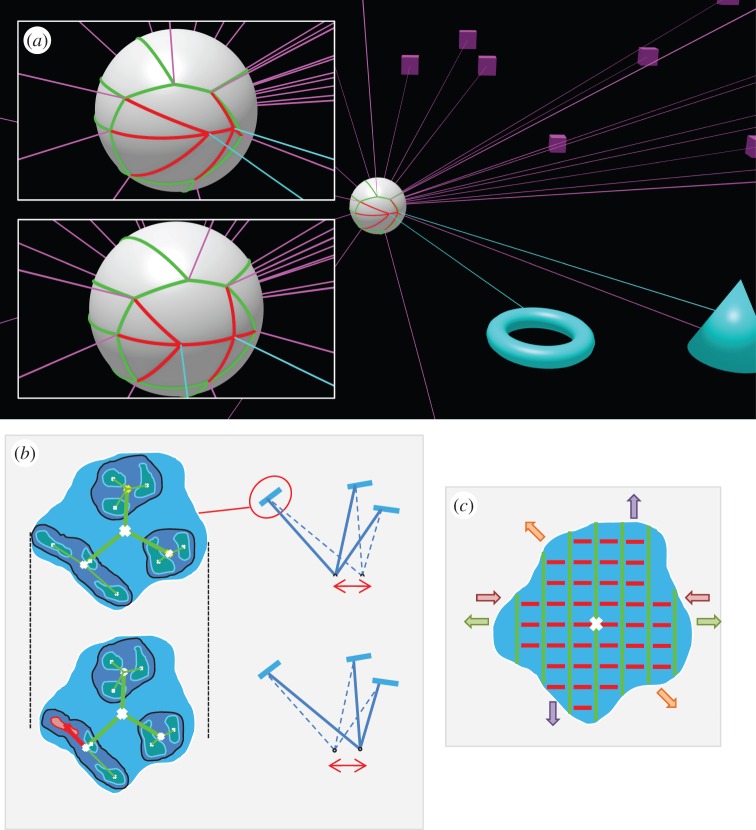
Image consequences of observer movement. Information about the distance, slant and local shape of surfaces can be gathered from the propensity of features to move relative to one another in the optic array as the observer moves in different directions in a static scene. (*a*) Static distant objects (in this case, purple cubes) do not change their relative visual direction as the observer moves (green arcs) whereas the image of near objects (shown in cyan) change relative to distant objects and relative to each other (red arcs). The white sphere shows the optic array around an optic centre in the centre of the sphere. The inset images show the optic array for two different locations of the optic centre (same static scene). Purple rays come from distant cubes, cyan rays come from near, cyan objects. The lengths of the arcs and the angles between them provide a description of the ‘relative visual direction’ of features in the optic array. For the green arcs, these remain stable despite reasonably large translations of the optic centre. (*b*) Considering a much smaller region of the optic array, a patch on a surface can also be considered in terms of features that remain constant despite observer movement versus those that change. The patch shown is slanted with respect to the line of sight and compresses horizontally as the observer translates. Most of the features compress in the same way as the overall compression of the patch (green arcs), as if drawn on a rubber sheet, whereas one feature moves (shown in red). It has depth relief relative to the plane of the surface patch [47]. (*c*) The tilt and qualitative information about the slant of the patch are indicated by the distortion of the rubber sheet. For any component of observer translation in a plane orthogonal to the line of sight (i.e. not approaching or backing away from the surface), different directions of observer movement produce effects shown by the coloured arrows (compression, red; expansion, green; shear, purple; a mixture, orange). The green rods stay the same length and remain parallel throughout; their orientation defines the tilt of the surface. The red lines joining the green rods indicate the ‘elasticity’ of the rubber sheet: the more elastic they are the greater the surface slant. Any component of observer translation towards the surface causes a uniform expansion of the whole sheet (including the green rods).

For example, consider a camera or eye rotating about its optic centre so that, over many rotations, it can view the entire panoramic scene or so-called optic array. If the axis and angle that will take the eye/camera from any point on this sphere to any other is recorded, then these relative visual directions provide a framework to describe the layout or ‘position’ of features across the optic array ([46]; figure 4*a*). In practice, the number of relative visual directions that need to be stored can be significantly reduced by organizing features hierarchically, e.g. storing finer scale features within coarser scale ones ([46,48,49], see figure 4*b*). This means that every fine-scale feature has a location within a hierarchical database of relative visual directions. Saccades allow the observer to ‘paint’ extra detail into the representation in different regions, like a painter adding brush strokes on a canvas, but the framework of the canvas remains the same [50].

When the optic centre translates (including the case of binocular vision, where the translation is from one eye to the other), information becomes available about the distance, slant and depth relief of surfaces:

— *Distance.* Some features in the optic array remain relatively stable with respect to each other when the optic centre translates [46]. Examples of these are shown in figure 4*a* (shown in green). For large angular separations, when pairs or triples of points do not move relative to one another in the face of optic centre translation, the points must be distant. These points form a stable background against which the parallax (or disparity) of closer features can be judged (shown in red in figure 4*a*). This type of representation of ‘planes plus parallax’ is familiar in computer vision, where explicit recovery of three-dimensional structure can be avoided, and many tasks simplified, by considering a set of points in a plane (sometimes these are points at infinity) and recording parallax relative to these points [51–53]. Representing the propensity of features to move relative to a stable background allows one to encode information about the relative distance of objects without necessarily forming a three-dimensional, world-based representation.
— *Depth relief.* Given that the definition of visual direction of features is recorded hierarchically in the proposed representation, there is a good argument for storing deformations in a hierarchical way, too. So, if a surface is slanted and translation of the optic centre causes a lateral compression of the image then the basis vector or coordinate frame for recording the visual direction of finer scale features should become compressed too. Koenderink and van Doorn [54] describe the advantages of using a ‘rubber sheet’ coordinate system like this. It has the effect that features on the slanted plane are recorded as having ‘zero’ disparity (or motion) and any disparity (or motion) signals a ‘bump’ on the surface (shown in red in figure 4*b*). There is good evidence that the visual system adopts a ‘rubber sheet’ coordinate frame of this sort from experiments on binocular correspondence [55], perceived depth [56] and stereoacuity [57,58].
— *Slant.* Figure 4*c* shows how the slant of a surface patch might be represented in a way that is short of a full metric three-dimensional description of its angle and yet useful for many purposes. Information about the image deformation of a surface patch provides information about the angle of tilt of the surface and some qualitative information about the magnitude of its slant (e.g. [59]). Figure 4*c* shows how moving in different directions is, in image terms, a bit like pulling and pushing a rubber sheet that contains rigid parallel rods: the more slanted the surface, the more elastic the connection between the green rods (shown as red lines in figure 4*c*). The tilt of the rods indicates the tilt of the surface. The rods can change in length (and the whole patch with them) if the optic centre moves towards or away from the surface.

**Figure 5.**
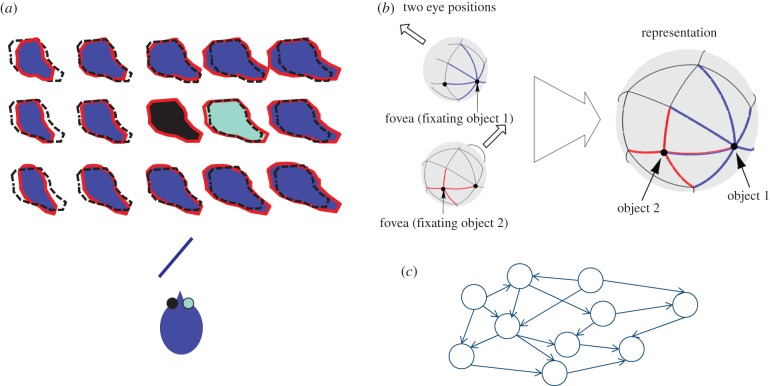
What might be stored? (*a*) An observer views a slanted surface, as shown in plan view. The image as seen from the left eye is shown in the centre (filled in black). The image immediately to the right (in cyan) shows how the right eye receives an image that is expanded laterally. The black dashed line shows the outline of the surface in the original (central) image. The top and bottom rows show the image consequences of the original (left eye) viewpoint moving up or down respectively and the columns show the consequences of the viewpoint moving left or right. These transformations can be understood in relation to a stable three-dimensional structure of the surface or in terms of the ‘propensity’ of parts of the image to change when the viewpoint moves. (*b*) An eye is shown viewing a static scene and rotating about its optic centre. The image from one viewing direction (shown in blue) overlaps with the image from another viewing direction (shown in red) and both these images can be understood in relation to a stable two-dimensional sphere of visual directions, as shown on the right. (Reprinted from Glennerster *et al.* [46] with permission from Elsevier.) (*c*) As discussed in the text, both these concepts (‘propensity’ and a stable ‘canvas’ on which to paint images) might be implemented using a graph where the nodes are sensory states and the edges are actions (translations of the head in (*a*) or rotations of the eye in (*b*)).

Thus, for distance, slant and depth relief we have identified information about the propensity of two-dimensional image quantities to change in response to observer movement (or binocular viewing). It is important to emphasize that this ‘propensity’ to change is neither optic flow [59,60] nor a three-dimensional reconstruction but somewhere in between. Figure 5 illustrates the point. Viewing a slanted surface gives rise to different optic flow depending on the direction of translation of the optic centre (figure 5*a*) but if the visual system is to use the optic flow generated by one translation to predict the flow that will be generated by a different head movement then it must infer something general about the surface. This need not be the three-dimensional structure of the surface, although clearly that would be one general description that would support predictions. Instead, the visual system might store something more image-based, as illustrated in figure 4. Neurally, this could be instantiated as a graph of sensory states joined by actions [45,61,62]. For example, if all the images shown in figure 5*a* correspond to nodes in a graph (figure 5*c*) and translations of the optic centre correspond to the edges, then the relationship between the nodes and the edges carries information about the surface slant: if a relatively large translation is required to move between nodes (the ‘propensity’ of the image to change with head translation is relatively low), then the surface slant is shallow. The same type of graph could underlie the idea of a ‘canvas’ on which to ‘paint’ visual information as the eyes move (figure 5*b*), as discussed above. The edges in this case are saccades [45,46,63–65].

Taken together, we now have a representation with many of the properties that Marr and Nishihara [66] proposed when they discussed a 2½D sketch. The eye can rotate freely and the representation is unchanged. Small translations in different directions (including from left to right eye, i.e. binocular disparity) give information about the relative depth of a surface, its slant relative to the line of sight and the relief of fine-scale features relative to the plane of the surface. It is, in a sense, an ego-centric representation in that the eye is at the centre of the sphere. Yet, in another sense, it is world-based, since distant points remain fixed in the representation, independent of the rotation and translation of the observer. This applies to an observer in a scene, moving their head and eyes, which is the situation Marr and Nishihara envisaged when they described their 2½D sketch. For larger translations, such as walking out of the room, a graph of views is still an appropriate representation [30] but many of the relationships illustrated in figure 4 would no longer apply for such ‘long baseline’ translations.

The purpose of Marr's primal sketch [67] and the 2½D sketch was that they were summaries, where information was made explicit if it was useful to the observer. That is also a feature of the representation described here, in that information can be left in ‘summary form’ or filled in in greater detail when required. In the case of an eye/camera rotating about its optic centre, there is ‘room’ in the representation to ‘paste in’ as much fine-scale detail as is available to the visual system [68], even though, under most circumstances, observers are unlikely to need to do this and, as many have argued, fine-scale detail need not be stored in a representation if it can be accessed readily by a saccadic eye movement when required (‘assuaging epistemic hunger’ as soon as it arises [69]).

Similarly, there is nothing to stop the visual system using the information from disparity or motion in the representation in a more sophisticated and calibrated way than simply recording measures such as ‘elasticity’ between features as outlined above. For example, it has been proposed that there is a hierarchy of tasks using disparity information [70,71] which goes from breaking camouflage at the simplest level through threading a needle, determining the bas-relief structure of a surface, comparing the relief of two surfaces at different distances to, at the top of the hierarchy, judging the Euclidean shape of a surface. These tasks lie on a spectrum in which more and more precise information is required, either about the disparities produced by a surface or about its distance from the observer, in order to carry out a task successfully. Judgement of Euclidean shape demands a calibration of disparity information—a precise delineation of the current sensory context relative to others—to such an extent that performance is compatible with the brain generating a full Euclidean representation. What is important in this way of thinking, though, is that the top level of calibration of disparities is only carried out when the task demands it (which is likely to be rare and, in the example in §3, occurs only once). If the visual system never used ‘summaries’ or short-cuts, then the storage of disparity and motion information would be equivalent to a full metric model of the environment.

## 3. Examples

### (a) An example task

As we said at the outset, for a representation to be useful to a moving observer, information gained at one location must be capable of guiding action at another. Consider a task: a person has to retrieve a mug from the kitchen, starting from the dining room. How can this be achieved, unless the brain stores a three-dimensional model of the scene? The first step is to rotate appropriately, which requires two things. The visual system must somehow know that the mug is behind one door rather than another, even if it does not store the three-dimensional location of the mug. In computer graphics applications, ‘portals’ are used in a related way to upload detailed information about certain zones of the virtual environment only when required [72]. The problem of knowing whether an item is down one branch or another of a deep nested tree structure is well known in relational databases [73] but is not considered here. Second, the direction and angle of the kitchen door relative to the current fixated object must be stored in the representation since it is currently out of view. Next, the person must pass through the door and rotate again so as to bring the mug into view. Each of these states and transitions could be considered as nodes and edges in a graph, with fairly simple actions joining the states.

The final parts of the task require a different type of interaction with the world, because the person must reach out and grasp the mug. The finger and thumb must be separated by an appropriate amount to grasp the mug and there is good evidence that information about the metric shape of an object begins to affect the grasp aperture before the hand comes into view [74–76]. It is possible that information from a number of sources, both retinal and extra-retinal, about the distance and metric structure of the target object can be brought to bear just at this moment (because, once the hand is in view, closed loop visual guidance can play a part [23,76–78]). As discussed above, the fact that a range of sources of relevant information can be brought to bear at a critical moment (when shaping the hand before a grasp) makes it very difficult to devise an experimental test that could distinguish between the predictions of competing models, i.e. a graph-based representation versus one that assumes the mug, hand and observer's head are all represented in a common three-dimensional reference frame with the shape of the mug described in full, Euclidean, metric coordinates.

That may seem slippery, from the perspective of designing critical experiments, but it is an important point. A graph of contexts connected by actions is a powerful and flexible notion. If the task is only to discriminate between bumps and dips on a surface, then the contexts that need to be distinguished can be very broad ones (e.g. positive versus negative disparities). On the other hand, the context corresponding to a mug with a particular size, shape and distance is a lot more specific. It may require not only more specific visual information but also information from other senses such as proprioception, including vergence, in order to narrow it down. All the same, it remains a context for action. During the mug-retrieving task, most of the steps do not require such a narrow, richly-defined context including all the stages at which visual guidance is possible or the action is a pure rotation of the eye and head. But some do, and in those cases it is possible to specify more precise, multi-sensory contexts that will discriminate between different actions.

### (b) Example predictions

When putting forward their stereo algorithm, Marr and Poggio [79] went to admirable lengths to list psychophysical and neurophysiological results that, if they could be demonstrated, would falsify their hypothesis. Here are a few that would make the proposals described above untenable (§2d). Using Marr and Poggio's convention, the number of stars by a prediction (*P*) indicates the extent to which the result would be fatal and *A* indicates supportive data that already exist.

**Figure 6.**
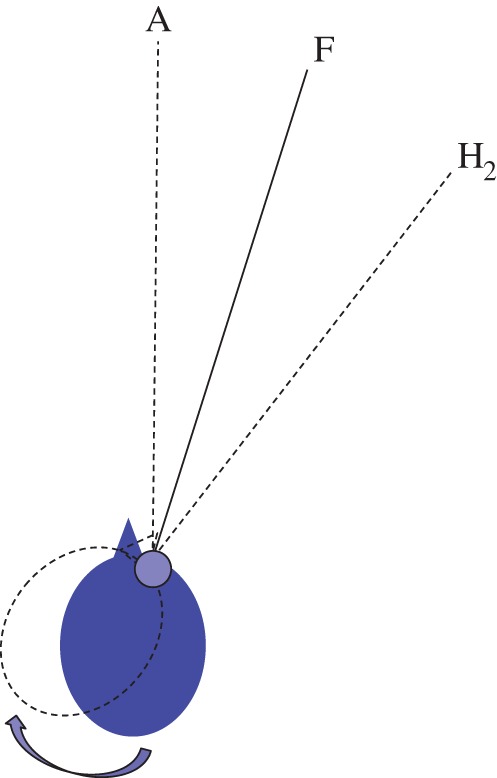
Potential evidence of head-centric adaptation. If a participant were to fixate a point F and adapt to a stimulus (e.g. a drifting grating) presented at A in the head-centric midline then turn their head to point in the direction H_2_ and test the effect of adaptation over a wide range of test directions, the predictions of retinotopic, spatiotopic and head-centric adaptation would differ. In this case, the peak effects should occur at A for retinotopic and spatiotopic adaptation and at H_2_ for head-centric adaptation. (Online version in colour.)

— (P***) *Coordinate transformations.* Strong evidence in favour of true coordinate transformations of visual information in the parietal cortex or hippocampus would be highly problematic for the ideas set out in §2d. If it could be shown that visual information in retinotopic visual areas like V1 goes through a rotation and translation *‘en masse’* to generate receptive fields with a new origin and rotated axes in another visual area, where these new receptive fields relate to the orientation of the head, hand or body then the ideas set out in §2d will be proved wrong, since they are based on a quite different principle. Equally fatal would be a demonstration that the proposal illustrated in figure 3*b* is correct, or any similar proposal involving multiple duplications of a representation in one coordinate frame in order to choose one of the set based on idiothetic information. Current models of coordinate transformations in parietal cortex are much more modest, simulating ‘partially shifting receptive fields’ [80] or ‘gain fields’ [38] which are two-, not three-dimensional transformations. Similarly, models of grid cell or hippocampal place cell firing do not describe how three-dimensional transformations could take place taking input from visual receptive fields in V1 and transforming them into a different, world-based three-dimensional coordinate frame [81–83].
— (P***) *World-centred visual receptive fields.* This does not refer to receptive fields of neurons that respond to the location of the observer [84]. After all, the location of the observer is not represented in any V1 receptive field (it is invisible) so no rotation and translation of visual receptive fields from retinotopic to egocentric to world-centred coordinates could make a place cell. A world-centred visual receptive field is a three-dimensional ‘voxel’ much like the three-dimensional receptive field of a disparity-tuned neuron in V1 but based in world-centred coordinates. Its structure is independent of the test object brought into the receptive field and independent of the location of the observer or the fixation point. For example, if the animal viewed a scene from the south and then moved, in the dark, round to the west, evidence of three-dimensional receptive fields remaining constant in a world-based frame would be incompatible with the ideas set out here. In this example, the last visual voxels to be filled before the lights went out should remain in the same three-dimensional location, contain the same visual information (give or take general memory decay across all voxels) and remain at the same resolution, despite the translation, rotation and new fixation point of the animal. An experiment that followed this type of logic but for pointing direction found, on the contrary, evidence for gaze-centred encoding [85].
— (A*) *Task-dependent performance.* If all tasks are carried out with reference to an internal model of the world (a ‘cognitive map’ or reconstruction), then whatever distortions there are in that model with respect to ground truth should be reflected in all tasks that depend on that model. Proof that this is the case would make the hypothesis set out in §2d untenable. However, there is already considerable evidence that the internal representation used by the visual system is something much looser and, instead, that different strategies are used in response to different tasks. Many examples demonstrate such ‘task-dependence’ [71,86–89]. For example, when participants compare the depth relief of two disparity-defined surfaces at different distances they do so very accurately while, at the same time, having substantial biases in depth-to-height shape judgements [71]. This experiment was designed to ensure that, to all intents and purposes, the binocular images the participant received were the same for both tasks so that any effect on responses was not due to differences in the information available to the visual system. The fact that biases were systematically different in the two tasks rules out the possibility that participants were making both judgements by referring to the same internal ‘model’ of the scene. Discussing a related experiment that demonstrates inconsistency between performance on two spatial tasks, Koenderink *et al.* [86, p. 1473] suggest that it might be time to ‘… discard the notion of “visual space” altogether. We consider this an entirely reasonable direction to explore, and perhaps in the long run the only viable option’.
— (P**) *Head-centred adaptation.* A psychophysical approach could be, for example, to look for evidence of receptive fields that are constant in head-centred coordinates. For example, if an observer fixates a point 20° to the right of the head-centric midline and adapts to a moving stimulus 20° to the left of fixation (i.e. on the head-centric midline), do they show adaptation effects in a head-centric frame after they rotate their head to a new orientation while maintaining fixation (see figure 6)? Evidence of a pattern of adaptation that followed the head in this situation would not be expected according to the ideas set out in §2d. As figure 6 illustrates, this prediction is different from either retinal or spatiotopic (world-based) adaptation [90–92]. There is psychophysical evidence that gaze direction can modulate adaptation [93,94] consistent with physiological evidence of ‘gain fields’ in parietal cortex [38] but the data do not show that adaptation is spatially localized in a head-centred frame as illustrated in figure 6.

## 4. Conclusion

If a moving observer is to use visual information to guide their actions, then they need a visual representation that encodes the spatial layout of objects. This need not be three-dimensional, but it must be capable of representing the current state, the desired state and the path between the two. Computer vision representations are three-dimensional, predominantly, as are most representations that are hypothesised in primates but, I have argued, there is good reason to look for alternative types of representation that avoid three-dimensional coordinate frames.
